# Effector-Specific Characterization of Brain Dynamics in Manual vs. Oculomotor Go/NoGo Tasks

**DOI:** 10.3389/fnhum.2020.600667

**Published:** 2020-12-03

**Authors:** Marie Simonet, Paolo Ruggeri, Jérôme Barral

**Affiliations:** ^1^Institute of Sport Sciences, University of Lausanne, Lausanne, Switzerland; ^2^Brain Electrophysiology Attention Movement Laboratory, Institute of Psychology, University of Lausanne, Lausanne, Switzerland

**Keywords:** inhibitory control, electroencephalogram, topographic analysis of variance, source localization, precuneus

## Abstract

Motor inhibitory control (IC), the ability to suppress unwanted actions, has been previously shown to rely on domain-general IC processes that are involved in a wide range of IC tasks. Nevertheless, the existence of effector-specific regions and activation patterns that would differentiate manual vs. oculomotor response inhibition remains unknown. In this study, we investigated the brain dynamics supporting these two response effectors with the same IC task paradigm. We examined the behavioral performance and electrophysiological activity in a group of healthy young people (*n* = 25) with a Go/NoGo task using the index finger for the manual modality and the eyes for the oculomotor modality. By computing topographic analysis of variance, we found significant differences between topographies of scalp recorded potentials of the two response effectors between 250 and 325 ms post-stimulus onset. The source estimations localized this effect within the left precuneus, a part of the superior parietal lobule, showing stronger activity in the oculomotor modality than in the manual modality. Behaviorally, we found a significant positive correlation in response time between the two modalities. Our collective results revealed that while domain-general IC processes would be engaged across different response effectors in the same IC task, effector-specific activation patterns exist. In this case, the stronger activation of the left precuneus likely accounts for the increased demand for visual attentional processes in the oculomotor Go/NoGo task.

## Introduction

Inhibitory control (IC), the ability to suppress ongoing actions or to ignore distracting or irrelevant stimuli, is a key executive function enabling goal-directed behavior (Spierer et al., 2013; Aron et al., 2014; Hartmann et al., 2019). Neurophysiologically, previous literature has postulated the existence of domain-general IC mechanisms that support all inhibitory tasks (Spierer et al., 2013). It has also been advanced that depending on the task’s demands (Dillon and Pizzagalli, 2007; Simonet et al., 2019) or the response modality (Leung and Cai, 2007), more specific IC processes can be engaged.

The idea of a “global suppressive network” that would partly brake or stop motor activity and cognition was well-documented by Wessel and Aron (2017). The authors argued that global stopping mechanisms supported by a hyperdirect, fronto-basal-ganglia pathway are engaged when action errors or unexpected events occurred. The existence of a global “non-selective brake” that would suppress actions or at least delay motor responses was also proposed by Coxon et al. (2006, 2009) when investigating unimanual vs. bimanual selective inhibition (Coxon et al., 2007) and selective vs. non-selective inhibition (Coxon et al., 2009). In the same vein, Sallard et al. (2014) used a Go/NoGo task with global vs. selective inhibition stimuli and concluded that, while there exist global mechanisms underlying global and selective inhibition, both types of inhibition also relied on different neural engagement at an early processing stage.

If IC relies on global mechanisms, the literature suggests that the modulations induced by the regular practice of an IC task-sharing similar or close components with another cognitive task could lead to transfer to non-trained tasks (Verbruggen et al., 2012; Maraver et al., 2016). Maraver et al. (2016), for instance, compared the effectiveness of six working memory or IC training sessions by assessing the near and far transfer effects to various closely related cognitive tasks, including the Stroop task, the N-back task, the Stop-signal task, and the Operation Span task, among others. Overall, their results support the executive function “Unity and Diversity” model developed by Miyake et al. (2000) which shows that training a particular executive function (diversity) can lead to near and far transfer effects (unity) to untrained tasks. Specific to IC, they found a transfer effect to a close stop-signal task and far control strategy and abstract reasoning tasks following inhibition training. Recently, Simonet et al. (2019) investigated whether practicing a complex Go/NoGo task involving perceptual and task-set control components in addition to motor inhibition would lead to functional changes in IC areas and transfer effects to untrained tasks. Despite modulations within domain-general IC areas, the authors found no transfer effects, which suggests that different facets or subcomponents of IC rely on specific regions within this domain-general IC network (Dillon and Pizzagalli, 2007). While different IC tasks have been investigated to clarify the organization of this network and its subcomponents (Dillon and Pizzagalli, 2007; Spierer et al., 2013; Liu et al., 2016; Chavan et al., 2017; Rey-Mermet et al., 2018; Simonet et al., 2019), whether specific IC brain regions support different response effectors remains unknown.

To date, the neural mechanisms underlying manual and oculomotor inhibition have been widely studied with a range of different tasks and neuroimaging methods, such as electroencephalogram (EEG), functional magnetic resonance, or magnetoencephalography. However, only one study has previously compared manual and oculomotor response inhibition within the same task (Leung and Cai, 2007). Interestingly, the authors found some overlapping activations within the ventrolateral prefrontal cortex but also more specific processes supporting manual and oculomotor response inhibition. To further investigate the inhibition processes supporting both motor responses, we decided to design another inhibitory task with the same two response effectors: the hand and the eyes. Our experimental protocol thus combined two Go/NoGo tasks and electrical neuroimaging methods to disentangle the domain-general IC processes and understand their subcomponents and specificities. Most of the studies investigating the neural networks underlying IC have used either one response effector in one IC task (Manuel et al., 2013; Hartmann et al., 2016; Chavan et al., 2017), one response effector in a set of different IC tasks (Enge et al., 2014; Maraver et al., 2016), or two response effectors (manual and oculomotor) in two different IC tasks (Connolly et al., 2000; Chikazoe et al., 2007), or they have merged different tasks into one single IC task (Enriquez-Geppert et al., 2010; Scharinger et al., 2015; Simonet et al., 2019). However, there are only a few direct comparisons between manual and oculomotor response effectors within the same task (Leung and Cai, 2007). To the best of our knowledge, this study is the first attempt to investigate the neural processes underlying manual inhibition and oculomotor inhibition with the same Go/NoGo paradigm.

Regarding the timing and localization of IC processes, previous works investigating IC processes using electroencephalography (EEG), have revealed neural correlates of inhibitory mechanisms over fronto-central sites in the N2/P3 complex. This complex includes early (N2: 200–300 ms) and late (P3: 300–500 ms) phases of inhibition processes (Kok et al., 2004; Smith et al., 2006; Vuillier et al., 2016). In studies using Go/NoGo tasks with manual responses, these processes have been principally located within the right inferior frontal cortex, the pre-supplementary motor area, and anterior cingulate (Bokura et al., 2001; Aron et al., 2014; Baumeister et al., 2014; Sallard et al., 2014; Chavan et al., 2015; Hartmann et al., 2016), and the left lateral and medial prefrontal regions (Rubia et al., 2001; Swick et al., 2008; Simonet et al., 2019). For oculomotor inhibition, studies using anti-saccade tasks mainly localized inhibition processes in the anterior cingulate cortex, the inferior frontal cortex, the pre-supplementary motor area, the frontal eye field, and regions in the inferior and superior parietal cortex (Heinen et al., 2006; Chikazoe et al., 2007; McDowell et al., 2008; Neggers et al., 2012; Jamadar et al., 2013; Herweg et al., 2014; Talanow et al., 2018). Finally, beyond the diversity of the task paradigms presented in the literature, the diversity of the methods used to analyze the data [event-related potential (ERP) analyses, frequency-domain EEG analyses, and microstate analyses, among others] requires caution when predicting the brain regions involved in specific IC tasks.

In the present study, we examined the IC mechanisms supporting manual and oculomotor modalities in the same IC task. Participants performed a Go/NoGo task with two different response effectors: the index finger for the Go/NoGo manual modality and the eyes for the Go/NoGo oculomotor modality. Brain dynamics were explored by recording EEG during task execution. To investigate the entire time course of inhibition processes, we performed time-wise topographic analyses and electrical source estimations when participants had to withhold their motor response. Capitalizing on previous findings (Smith et al., 2006; Hartmann et al., 2016; Vuillier et al., 2016), we hypothesize that the expected topographical differences between the two response effectors would appear in the N2/P3 complex between 200 and 400 ms. Regarding the source estimation analyses, we expect to find differences in the ventrolateral prefrontal cortex (Buchsbaum et al., 2005; Leung and Cai, 2007) and inferior and superior parietal regions (McDowell et al., 2008; Herweg et al., 2014). As complementary EEG analyses and to provide insight into the specific brain regions activated in the Go condition, we will compute the same analyses for the Go condition and the NoGo condition. Since our participants responded with the right hand, we expect to find specific activation in the left sensorimotor and premotor cortices in the manual modality compared to the oculomotor modality (Witt et al., 2008; Olman et al., 2012). Furthermore, we assumed that the frontal eye field would be more involved in the oculomotor modality than the manual modality, as this region is commonly known to control voluntary saccades (Vernet et al., 2014).

## Materials and Methods

### Participants

We included 25, right-handed healthy adults in this study based on previous EEG literature on IC and literature performing identical EEG analyses (Egenolf et al., 2013; Hartmann et al., 2016, 2019). The participants reported normal or corrected-to-normal vision and no history of neurological or psychiatric disease. Six participants were excluded from the analyses due to a bad EEG signal (*n* = 5) or misunderstanding of the tasks’ instructions (*n* = 1). Nineteen participants (4 females; 15 males; mean age ± SD = 25.5 ± 3) were included in the analyses. The experimental protocol was approved by the Cantonal Ethics Committee for Human Research (Vaud, Switzerland; protocol N° 419/15).

### Experimental Procedure

Participants were seated in a dark, quiet room facing a computer screen (Dell, 1707FPt 17^′′^ Flat Panel Monitor, TX, USA) placed 50 cm from their eyes. They completed two Go/NoGo tasks involving a manual and an oculomotor modality. The two tasks were randomly performed one after another to avoid confusion between the instructions so that half of the participants performed the manual Go/NoGo task first and then the oculomotor Go/NoGo task, and half of the participants performed the oculomotor Go/NoGo task first and then the manual Go/NoGo task. Stimulus delivery was controlled using E-Prime 2.0 software (Psychology Software Tools Inc., Sharpsburg, PA, USA).

**Figure 1:**
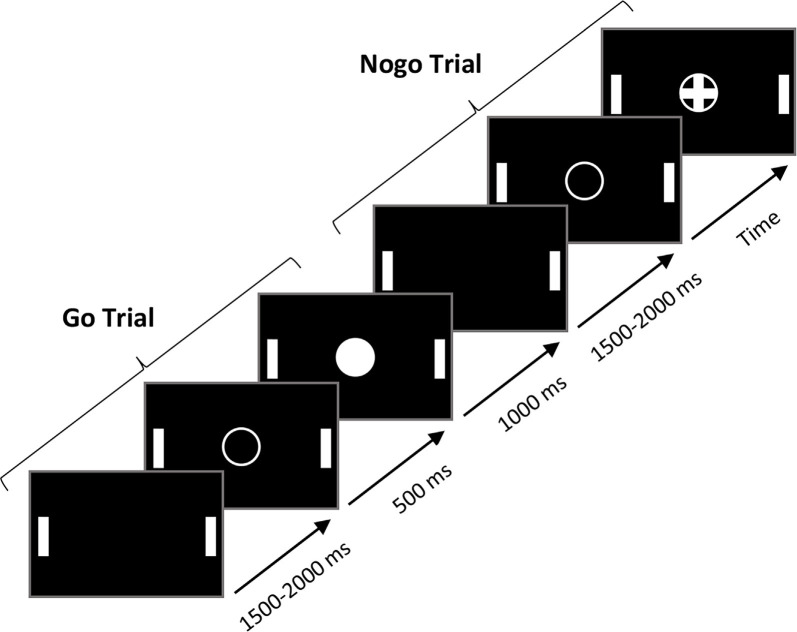
The paradigm for the Go/NoGo tasks. Go trials and NoGo trials were the same in the manual and oculomotor modality.

The manual Go/NoGo task required participants to press, as fast as possible, a QWERTY keyboard’s spacebar with their right index finger when Go stimuli appeared while suppressing their motor response to NoGo stimuli. The response time (RT) was automatically recorded by the E-prime 2.0 software. A trial started with an empty white circle displayed centrally on a black screen for 500 ms. Then, a cross in a circle (the NoGo stimulus) or a filled circle (the Go stimulus) appeared in random order for 1,000 ms. The next trial started after a time interval ranging from 1,500 to 2,000 ms (see Figure 1 for the paradigm for the Go/NoGo tasks). In total, participants performed five blocks of 60 trials each, with a stimulus probability of 0.3 and 0.7 for the NoGo and Go stimuli, respectively (210 Go and 90 NoGo stimuli in total). Before the experimental session, participants completed a “calibration” phase of 20 trials (13 Go, 7 NoGo) to estimate their average RT to Go stimuli (for a similar procedure, see Simonet et al., 2019). An RT threshold, corresponding to 90% of the mean RT of the calibration phase, was then computed. During the five experimental blocks, a feedback “faster” was displayed on the screen when the participant’s RT was above the RT threshold. This feedback was presented to encourage participants to respond faster. IC performance was indexed by the RT to Go stimuli and the percentage of errors to NoGo stimuli (false alarms, FA). RTs <100 ms and >2 standard deviations of the individual’s mean RT were excluded from the analysis.

For the oculomotor Go/NoGo task, the task paradigm (i.e., number of trials, the ratio of Go/NoGo trials, time course of the experiment, etc.) was the same as the manual Go/NoGo task. To assess the oculomotor response, participants were required to make a volitional saccade towards the right target (a vertical white stick) as quickly as possible and return to the center of the screen each time a Go stimulus appeared while staring at the center of the screen when NoGo stimuli appeared. Note that white sticks also appeared in the manual Go/NoGo task to ensure it was the same visual environment. Two electrodes placed on the temples close to the eyes were used to record the horizontal eye movements, and the RT was computed *via* these two electrodes. For each participant in the Go condition, we averaged the peak values of the signal (in μV) when the eyes reached the target on the right and then calculated a threshold that represented 30% of this peak’s average. We considered the time delay between stimulus onset and this threshold corresponded to the oculomotor RT. In NoGo trials, a saccade exceeding this threshold was considered an error.

### Behavioral Analyses

Mean RTs and FA rates obtained from the execution of the manual and oculomotor Go/NoGo tasks are presented without statistical comparison as the method to extract these variables was not consistent between the two response effectors. However, to demonstrate the within-subject association between the manual and oculomotor modalities, we performed Pearson correlation analyses with the manual and oculomotor RT and the manual and oculomotor FA rate. Statistical significance was set at *p* < 0.05.

### EEG Recording and Preprocessing

EEG data were recorded with a 64-channel Biosemi ActiveTwo amplifier system (Biosemi, Amsterdam, Netherlands) at a sampling rate of 2,048 Hz and processed using Brain Vision Analyzer (Version 2.0.1.391; Brain Products, Munich, Germany). Data were combined with stimulus delivery using E-prime 2.0 and automatically synchronized with markers in the continuous EEG file.

After 512 Hz downsampling and filtering (0.31–40 Hz bandpass), eye movement artifacts were corrected using independent component analysis (ICA; Cardoso, 1998). Electrodes containing artifacts were interpolated using 3D splines (Perrin et al., 1987) leading to an average of 4.5% of interpolated electrodes across all participants (SD = 3.0; max = 9.4%, min = 0%). ERPs were segmented from 100 ms pre-stimulus to 400 ms post-stimulus onset separately for successful Go and NoGo trials. After segmentation, the epochs were cleaned by applying a semiautomatic procedure using a ±80 μV artifact rejection criterion and by visually inspecting the signal epoch-by-epoch. We manually removed the epochs containing eye blinks or motor artifacts. Go and NoGo epochs were averaged separately. In total, the average number of accepted epochs ± standard deviation was 195.3 ± 14.6 for manual Go (mean: 7.0% rejection), 71.6 ± 12.6 for manual NoGo (mean: 19.7% rejection), 201.8 ± 14.2 for oculomotor Go (mean: 3.9% rejection), and 69.1 ± 10.1 for oculomotor NoGo (mean: 23.2% rejection) trials. The data were finally recalculated to an average reference. No baseline correction was applied.

### EEG Analyses

#### Tests of Topographic Consistency Between Subjects

We first assessed the topographic consistency of the ERPs of each response effector to identify periods with consistent patterns of source activity across subjects. This process was done using the topographic consistency test (TCT), which helps identify periods where there is evidence of a consistent relation between an event and the underlying brain source generators. This test was necessary to avoid false conclusions resulting from an incorrect selection of time windows of interest (Koenig and Melie-García, 2010). The TCT was implemented using the open-source software, RAGU (Randomization Graphical User interface; Koenig et al., 2011) in MATLAB^1^. We applied a TCT to the ERPs of each response effector (i.e., manual and oculomotor) over this time interval to check whether this period showed a consistent pattern of active sources across subjects (Koenig and Melie-García, 2010). The TCT was computed with 10,000 randomization runs and a *p* threshold of 0.01. To control for multiple testing, we performed a global duration statistics test and considered only continuous periods of significance (Koenig and Melie-García, 2010; Habermann et al., 2018). This process was used to test whether the pattern of a significant period exceeded chance (for a detailed procedure, see Ruggeri et al., 2019).

#### Topographic Differences Analyses

To identify the periods of significant topographic differences between conditions (Strik et al., 1998; Murray et al., 2008; Michel et al., 2009), we performed a topographic analysis of variance (TANOVA) from −100 ms to 400 ms post-stimulus onset with the open-source software, RAGU, in MATLAB. Using a nonparametric randomization test, this analysis assesses global dissimilarities in the whole electric field between conditions at each time point.

The TANOVA was computed on amplitude-normalized maps [Global field power (GFP) = 1] to obtain results that are independent of global field strength. We performed this normalization because we were interested in the significant spatial differences between the response effectors (i.e., manual and oculomotor) and not in the differences in strength of similar source distributions between the effectors. The two response effectors were compared for the Go and NoGo conditions separately. The analyses were computed at each time point, with 10,000 randomization runs and a *p* threshold of 0.01. To control for multiple tests, we performed the global duration statistics test and considered only continuous periods of significance (Koenig and Melie-García, 2010; Koenig et al., 2011; Habermann et al., 2018). This process was used to test whether the differences in a significant period exceeded chance. After observing periods of interest (POI) above the duration threshold, a *post hoc* channel-wise *t*-test (*t*-maps) enabled further investigation of the topographic distribution of the observed differences between the manual and oculomotor modalities in the Go and NoGo conditions.

#### Electrical Source Estimations

Electrical source estimations were performed using the sLORETA method (Pascual-Marqui, 1999, 2002). sLORETA is a linear inverse imaging method that is calculated by standardizing a minimal norm inverse solution by source variance and measurement noise (see Pascual-Marqui, 2002 for further details). The sLORETA solution space is made of 6,239 voxels and has a 5-mm spatial resolution and is restricted to the gray matter of cortical and hippocampal regions. To identify differences in the pattern of active cortical sources responsible for the topographic differences observed between the response effectors in both the Go and NoGo conditions, normalized ERPs (i.e., GFP = 1) were averaged over the POI identified with the TANOVA analyses before source localization. The resulting current source density data were compared between the response effectors in the Go and the NoGo conditions using nonparametric voxel-wise *t*-tests with 10,000 permutations. This procedure determined the critical probability threshold (*p* < 0.05, one-tailed) for the observed *t*-values with correction for multiple tests. All source estimations were performed with a signal-to-noise ratio of 100.

## Results

### Behavioral Data

**Figure 2:**
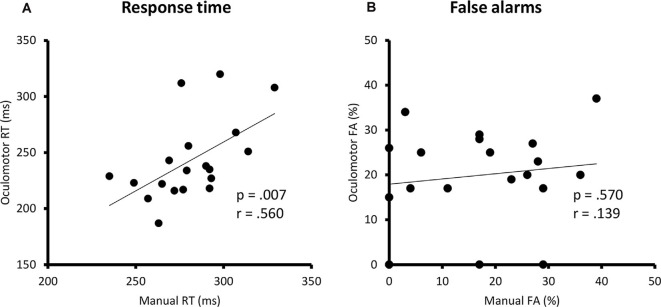
Correlation analyses between the manual and oculomotor RTs in ms **(A)** and between the manual and the oculomotor FA in percentage **(B)**. Linear regression lines, *p*-values, and Pearson correlation coefficients are represented. RT, response time; ms, milliseconds; FA, false alarm.

The Pearson correlation analyses revealed a significant positive correlation between the manual and the oculomotor modalities for the RTs [manual: 281 ± 22.8 ms (mean ± SD); oculomotor: 243 ± 36.2 ms; *r* = 0.560, *p* = 0.007] but not the FA rates (manual: 17 ± 12.6%; oculomotor: 20 ± 10.6%; *r* = 0.139, *p* = 0.570; Figure 2).

### Electrical Neuroimaging Results

#### TCT

The TCT was applied to the preprocessed ERPs computed from −100 ms before the onset of the stimulus to 400 ms after. The test showed significant and consistent topographies across the subjects within this time interval corroborated by a significant global duration test. The TANOVA analysis was thus performed between −100 and 400 ms.

#### Topographic Analyses

**Figure 3:**
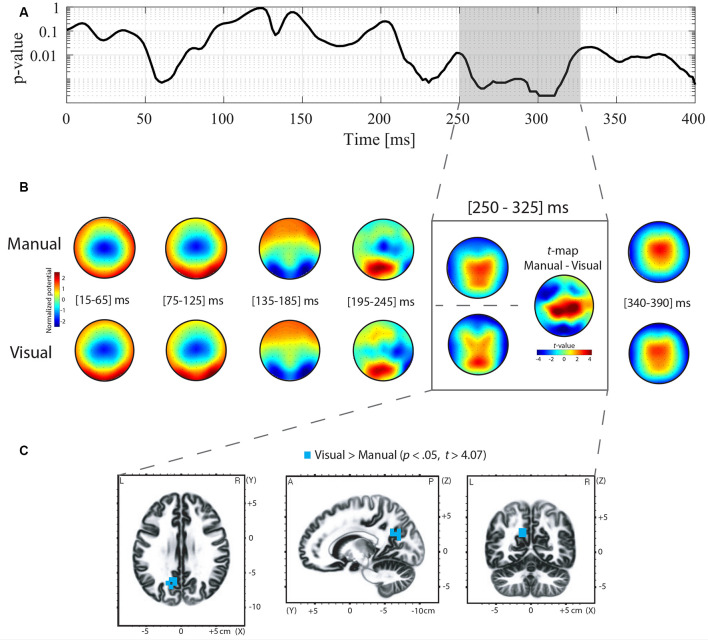
Topographic analysis of variance (TANOVA), averaged topographies, *t*-maps, and sLORETA analyses for the contrast between the manual and oculomotor modality for the NoGo condition. **(A)** The curve represents the *p*-values of the TANOVA plotted for each time point from 0 to 400 ms post-stimulus onset. The period of significant topographic differences is highlighted in gray. **(B)** Mean event-related potential (ERP) topographies were computed for the manual and oculomotor modalities separately over 50-ms time intervals. The topographies were normalized [Global field power (GFP) = 1]. Red represents positive potential values, whereas blue represents negative potential values. The *t*-map contrasting the manual and oculomotor modality is shown for the periods of interest (POI). Positive (in red) and negative (in blue) *t*-values indicate more positive and more negative potentials in the manual condition than in the oculomotor modality, respectively. **(C)** Voxel-wise *t*-values comparing the sLORETA source density between the oculomotor and the manual modality during the POI. Clusters of voxels located in the precuneus (BA 7, 31) showed increased activation in the oculomotor modality compared to the manual modality. All voxels reaching significance (*p* > 0.05, corrected for multiple comparison *t* > 4.07) are highlighted in yellow.

**Figure 4:**
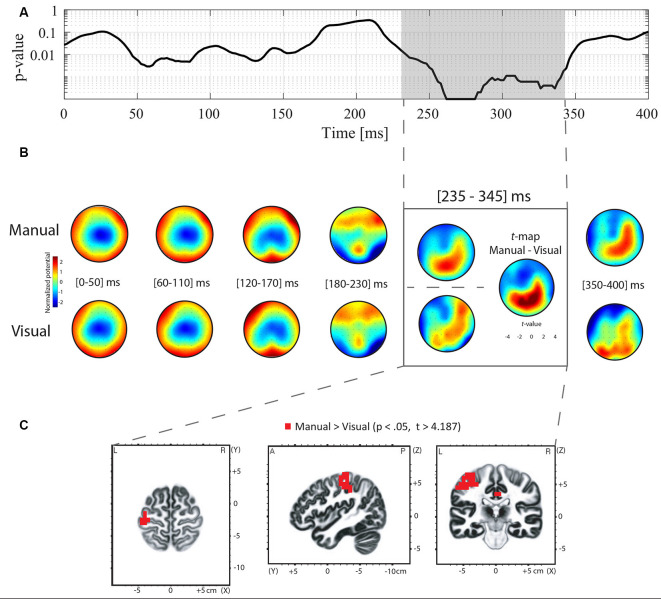
TANOVA, averaged topographies, *t*-maps, and sLORETA analyses for the contrast between the manual and oculomotor modality for the Go condition. **(A)** The curve represents the *p*-values of the TANOVA plotted for each time point from 0 to 400 ms post-stimulus onset. The period of significant topographic differences is highlighted in gray. **(B)** Mean ERP topographies were computed for the manual and the oculomotor modalities separately over 50-ms time intervals. The topographies were normalized (GFP = 1). Red represents positive potential values, whereas blue represents negative potential values. The *t*-map contrasting the manual and the oculomotor modality is shown for the POI. Positive (in red) and negative (in blue) *t*-values indicate more positive and more negative potentials in the manual condition than in the oculomotor modality, respectively. **(C)** Voxel-wise *t*-values comparing the sLORETA source density between the manual and the oculomotor modality during the POI. Clusters of voxels located in the postcentral gyrus (BA 1, 2, 3), the precentral gyrus (BA 4, 6), the cingulate gyrus (BA 23, 31), and the inferior parietal lobule (BA 40) showed increased activation in the manual modality compared to the oculomotor modality. All voxels reaching significance (*p* > 0.05, corrected for multiple comparison *t* > 4.187) are highlighted in red.

Differences in ERP topography between the manual and oculomotor modalities for the Go and NoGo conditions were examined using TANOVA analysis. For the NoGo condition, the TANOVA revealed significant topographic differences between the manual and oculomotor modalities between 250 and 325 ms post-stimulus onset (Figure 3A). In the Go condition, the TANOVA revealed significant topographic differences between the manual and oculomotor modalities between 235 and 345 ms post-stimulus onset (Figure 4A). The topographies of the two response effectors are displayed for the Go and NoGo conditions in Figures 3B, 4B, respectively.

To quantify these topographic effects, *t*-map contrasts were computed between topographies in the manual and oculomotor modalities from the periods with significant differences in the Go and NoGo conditions. In the NoGo condition (Figure 3B, gray frame), these contrasts revealed that the manual modality was characterized by a more positive potential over central electrodes and a more negative potential over occipital and left frontal electrodes (*t*_max_ = 7.078 at electrode CP1; *t*_min_ = −5.677 at electrode F5). In the Go condition (Figure 4B), the manual modality was characterized by a more positive potential over Centro-occipital electrodes (*t*_max_ = 6.863 at electrode P2; *t*_min_ = −4.726 at electrode Fz).

#### Electrical Source Estimations

sLORETA was applied in the periods of significant topographic differences found with the TANOVA (NoGo: 250–325 ms; Go: 235–345 ms) to estimate brain regions accounting for the observed topographic differences between the manual and oculomotor modalities. For the NoGo condition, the statistical analysis of normalized data demonstrated that the oculomotor modality produced higher activity in the left precuneus (parietal lobe, BAs 7, 31; *t* > 4.07 for a *p* < 0.05) than the manual modality (Figure 3C). The maximal *t*-value of the cluster (*t* = 4.26) was located in the left precuneus (BA 31, Talairach coordinates *x* = −15, *y* = −60, *z* = 30). For the Go condition, the statistical analysis of normalized data demonstrated that the manual modality produced higher activity within the left hemisphere in the postcentral gyrus (BA 1, 2, 3), the precentral gyrus (BA 4, 6), the cingulate gyrus (BA 23, 31), and the inferior parietal lobule (BA 40; *t* > 4.187 for a *p* < 0.05) compared to the oculomotor modality (Figure 4C). The maximal *t*-value of the cluster (*t* = 5.77) was located in the postcentral gyrus (BA 3, Talairach coordinates *x* = −45, *y* = −25, *z* = 65).

## Discussion

In this study, we compared the electrophysiological brain dynamics of manual vs. oculomotor inhibition using the same Go/NoGo task. Our results revealed greater involvement of the precuneus with the oculomotor modality compared to the manual modality at 250–325 ms post-stimulus onset when participants had to stop their action. Comparatively, regions including the postcentral gyrus, the precentral gyrus, the cingulate gyrus, and the inferior parietal lobule were more involved with the manual modality compared to the oculomotor modality at 235–345 ms post-stimulus onset when participants had to generate an action.

Behaviorally, our correlation analyses demonstrated a significant relationship between the manual and oculomotor modalities for RTs but not for the FA rate. According to previous research, RTs are the most sensitive dependent variable for measuring IC performance. Several studies have shown a decrease in RT after minutes or hours of IC training with no change in the FA rate (Manuel et al., 2013; Chavan et al., 2015; Hartmann et al., 2016; Simonet et al., 2019). When comparing with previous literature, our difference between the manual RT and the oculomotor RT is surprisingly small. First, the stimuli chosen (a cross in a circle for the NoGo stimulus and a filled circle for the Go stimulus) are relatively easy to discriminate compared to other manual Go/NoGo tasks using stimuli such as letters, different colors, shapes, images, et cetera. This choice of stimuli would explain the difference of RTs between our study (281 ms) and most of the studies using manual Go/NoGo tasks (between 350 and 400 ms). Second, most of the experiments investigating saccade and prosaccade latencies used eye-tracking systems that measure very accurate saccade latencies. Since, we did not use such a system, the oculomotor RTs found in this study and the saccadic latencies found in other studies can not be compared. This being said, it is noteworthy that no statistical comparison was computed between the two effectors in our study due to the different methods used to record the RT, the results of our significant positive correlations highlight a close relation between manual and oculomotor RT and thus provide evidence of some common inhibition ability across response effectors.

The timing of our significant differences in the NoGo trials, occurring between 250 and 325 ms post-stimulus onset, is consistent with previous literature showing that inhibitory processes typically occur in the N2/P3 complex, which is between 200–300 (N2) and 300–500 (P3) after stimulus onset (Baumeister et al., 2014; Simonet et al., 2019). For instance, Simonet et al. (2019) combined the Go/NoGo task paradigm with EEG recordings to define the periods in which IC processes occur. By computing ERP analyses, the authors identified these processes between 200 and 250 ms, which they associated with the detection and resolution of response conflict. Consistently, our between-effector difference takes place during the N2 component, which corresponds to the early phase of inhibition (Bokura et al., 2001; De Pretto et al., 2017; Simonet et al., 2019). This period has been shown to initiate inhibition processes (Falkenstein et al., 1999; De Pretto et al., 2017) and to reflect conflict and interference resolution (Millner et al., 2012; Gajewski and Falkenstein, 2013; Chmielewski and Beste, 2017). For the topography, the N2 component has been associated with frontocentral components reflecting unexpected stimulus mismatch and cognitive control and with more posterior scalp distribution that would be dependent on the response effector used in the task (Folstein and Van Petten, 2008). Folstein and Van Petten (2008) suggested that this posterior distribution was likely to be associated with the oculomotor modality and more generally with visual attention. This association between posterior N2 and visual attention was also shown in Senkowski and Herrmann (2002), who linked posterior N2 with the difficulty of visual discriminative tasks, and in Bokura et al. (2001), who related NoGo frontocentral N2 with visual stimulus modality. Our scalp topography results, i.e., a negative potential over frontal sites in both response effectors with more positive frontocentral topographies in the manual modality and more positive posterior topographies in the oculomotor modality within the N2 component, corroborated previous results showing that: (i) inhibitory processes take place during the N2 period; and (ii) the posterior positive scalp topographies found with the oculomotor modality would be related to an enhanced visual demand. Of note, the absence of differences around P3 could be due to the simple design of our Go/NoGo task that has led to faster RTs than the RTs found in previous literature (Hartmann et al., 2016; Simonet et al., 2019), and therefore, to earlier inhibitory processes. Additionally, this absence of a difference could be explained by the fact that we compared only successful NoGo trials because the inhibitory processes of successful and unsuccessful stop trials have been shown to differ in time and in their underlying brain areas (Kok et al., 2004).

The statistical analyses of the brain source estimations showed that the left precuneus was more activated in the oculomotor modality than in the manual modality. The precuneus is a portion of the posteromedial superior parietal lobe (SPL) and has been demonstrated to be part of a widespread network comprising associative cortical and subcortical structures (Cavanna and Trimble, 2006). Globally, the SPL has been functionally related to visual processing, visual attentional processes, and visual-spatial shifting (Dehaene et al., 2003; Cavanna and Trimble, 2006; Molenberghs et al., 2007). More specifically, the functional role of the precuneus, albeit complex, has been mainly associated with voluntary shifting attention for visual stimuli, episodic memory retrieval, visuospatial imagery, and when directing spatial attention during task execution, especially if two limbs are coordinated towards a unique trajectory (Lundstrom et al., 2005; Wenderoth et al., 2005; Cavanna and Trimble, 2006; Lévesque et al., 2006). Concerning inhibitory processes, Popov et al. (2018) investigated the brain network dynamics underlying IC with a color-word Stroop task and found that the precuneus was a key node within this IC network (Spielberg et al., 2015) and closely communicated with the middle and inferior parts of the frontal gyrus. Using a counting Stroop task, Fan et al. (2014) compared neurotypical children and children with ADHD and found less activation in the left SPL in ADHD children. They assumed that this decreased activation was linked to impaired visual processing. Additionally, the authors showed a positive correlation between the activation of the left SPL and the percentage of correct responses in a test of visual pattern recognition memory, which they related to better visual processing in neurotypical children. In the same vein, increasing activation of the left precuneus is correlated with the latency to a correct response in a visual processing task (Fan et al., 2017). Altogether, these results highlight the link between activation of the left precuneus and behavioral performance in visual processing tasks.

Given that our analysis performed at the scalp and source-level showed differences in the underlying distribution of source generators between the response effectors (without a specific difference in the GFP), we suggest that while similar regions are supporting both manual and oculomotor response inhibition (Leung and Cai, 2007), the precuneus is the region that would specifically support the oculomotor modality in our two Go/NoGo tasks, and this stronger activation could account for the increased demand on visual attentional processes. Nevertheless, since it has been shown that inhibiting saccades in the context of manual responses is resource-demanding (Huestegge and Koch, 2014), we could not exclude the possibility that the precuneus would be involved in a task requiring more inhibition. Futures studies should implement experimental protocols that modulate the inhibitory load in different Go/NoGo tasks to define the role of the precuneus in oculomotor inhibition more precisely.

For the Go condition, a first qualitative assessment of the topographic maps averaged within the time window that included the mean manual RT (i.e., 235–345 ms post-stimulus onset) corroborated previous findings showing a posterior positivity moving towards the anterior right direction (Schiller et al., 2016; Ruggeri et al., 2019). Moreover, the source estimation contrasts revealed differences in left-lateralized regions, including the postcentral gyrus, the precentral gyrus, the cingulate gyrus, and the inferior parietal lobule. Since participants were required to respond with their right index finger, finding activation within left-lateralized regions is coherent with previous literature showing activation in contralateral sensorimotor cortices, premotor cortex, and inferior parietal cortices when performing finger tapping tasks (Witt et al., 2008; Olman et al., 2012). This coherence highlights the accuracy and robustness of our methods, and therefore, of the results presented for the NoGo condition.

While we highlighted the scalp topographies and the regions that would specifically support the oculomotor modality compared to the manual modality, the need to better disentangle the neural correlates underlying motor IC when using different response effectors within the same task paradigm calls for further investigations. Also, it would be interesting to assess manual and oculomotor inhibition with other inhibition tasks, such as the Eriksen Flanker task or the stop-signal task. Comparing the manual vs. oculomotor modalities in different inhibition tasks would provide further evidence of whether the precuneus generally supports oculomotor inhibition. We recommend that researchers seek to separately manipulate the specific subcomponents of this domain-general IC network using different response effectors in different IC tasks to implement practice-based research protocols at a later stage. Finally, because the white sticks appeared in the oculomotor condition and in the manual condition, one might wonder whether the eyes would have been attracted by this visual stimulus in the manual condition and thereby would have moved in the direction of the white sticks. If so, the manual condition could be considered as a dual-response condition. In the future and due to the possibility of dual-response phenomena, it would be interesting to capture eye movements with a high-resolution eye tracker. Beyond its fundamental relevance in the field of cognitive neuroscience, using the oculomotor effector represents a suitable motor effector to evaluate IC and to activate this IC network among patients with upper-limb motor disabilities (Bissett et al., 2015; Federico and Perez, 2017; Ganos et al., 2018; Lucci et al., 2019). The benefits of enhancing motor control in more global interventions that combine exercises with Go and NoGo situations are unknown.

Our electrical neuroimaging analyses have provided novel evidence concerning the brain regions differentially involved in manual and oculomotor response inhibition. The two strengths of this study lie in: (i) the implementation of a unique Go/NoGo task performed with two motor response effectors; and (ii) the computation of advanced EEG statistical analyses. Regarding the experimental design, only one study has been previously conducted with one unique inhibition task, a stop-signal task, performed with two motor effectors and accompanied by neuroimaging methods (Leung and Cai, 2007). Consistent with our results, this study presented evidence of partially separated IC processes supporting the hand motor and oculomotor inhibitory systems.

In summary, using the same Go/NoGo task with two response effectors, we showed for the first time that the left precuneus was more involved in the oculomotor modality than in the manual modality. The increased demand for visual attentional processes in the oculomotor Go/NoGo task would explain the stronger activation in this specific region of the SPL. Beyond its methodological rigor, this study framed some important mechanisms that are essential for better understanding the neural processes underlying IC. Overall, while similar domain-general IC mechanisms would be engaged across a wide variety of IC tasks and response modalities (Spierer et al., 2013; Simonet et al., 2019), there are specific regions or subregions of this domain-general IC network that would support specific response effectors.

## Data Availability Statement

The raw data supporting the conclusions of this article will be made available by the authors, without undue reservation.

## Ethics Statement

The studies involving human participants were reviewed and approved by the Cantonal Ethics Committee for Human Research (Vaud, Switzerland; protocol N° 419/15). The patients/participants provided their written informed consent to participate in this study.

## Author Contributions

MS acquired the data and wrote the main manuscript. MS and PR pre-processed and analyzed the EEG data, prepared the figures for the EEG data. MS, PR, and JB interpreted the EEG data. MS, PR, and JB interpreted the behavioral data. All authors conceived and designed the study. All authors contributed to the article and approved the submitted version.

## Conflict of Interest

The authors declare that the research was conducted in the absence of any commercial or financial relationships that could be construed as a potential conflict of interest.

## Footnotes

^1^http://www.mathworks.com/

## References

[B1] Aron A. R. Robbins T. W. Poldrack R. A. (2014). Inhibition and the right inferior frontal cortex: one decade on. Trends Cogn. Sci. 18, 177–185. 10.1016/j.tics.2013.12.003 24440116

[B2] Baumeister S. Hohmann S. Wolf I. Plichta M. M. Rechtsteiner S. Zangl M.. (2014). Sequential inhibitory control processes assessed through simultaneous EEG-fMRI. NeuroImage 94, 349–359. 10.1016/j.neuroimage.2014.01.023 24473101

[B3] Bissett P. G. Logan G. D. van Wouwe N. C. Tolleson C. M. Phibbs F. T. Claassen D. O.. (2015). Generalized motor inhibitory deficit in Parkinson’s disease patients who freeze. J. Neural Transm. 122, 1693–1701. 10.1007/s00702-015-1454-9 26354102 PMC4644440

[B4] Bokura H. Yamaguchi S. Kobayashi S. (2001). Electrophysiological correlates for response inhibition in a go/nogo task. Clin. Neurophysiol. 112, 2224–2232. 10.1016/s1388-2457(01)00691-5 11738192

[B5] Buchsbaum B. R. Greer S. Chang W.-L. Berman K. F. (2005). Meta-analysis of neuroimaging studies of the wisconsin card-sorting task and component processes. Hum. Brain Mapp. 25, 35–45. 10.1002/hbm.20128 15846821 PMC6871753

[B6] Cardoso J.-F. (1998). Blind signal separation: Statistical principles. Proc. IEEE 86, 2009–2025. 10.1109/5.720250

[B7] Cavanna A. E. Trimble M. R. (2006). The precuneus: a review of its functional anatomy and behavioural correlates. Brain 129, 564–583. 10.1093/brain/awl004 16399806

[B8] Chavan C. F. Mouthon M. Draganski B. van der Zwaag W. Spierer L. (2015). Differential patterns of functional and structural plasticity within and between inferior frontal gyri support training-induced improvements in inhibitory control proficiency. Hum. Brain Mapp. 36, 2527–2543. 10.1002/hbm.22789 25801718 PMC6869523

[B9] Chavan C. Mouthon M. Simonet M. Hoogewoud H.-M. Draganski B. van der Zwaag W.. (2017). Sustained enhancements in inhibitory control depend primarily on the reinforcement of fronto-basal anatomical connectivity. Brain Struct. Funct. 222, 635–643. 10.1007/s00429-015-1156-y 26659646

[B10] Chikazoe J. Konishi S. Asari T. Jimura K. Miyashita Y. (2007). Activation of right inferior frontal gyrus during response inhibition across response modalities. J. Cogn. Neurosci. 19, 69–80. 10.1162/jocn.2007.19.1.69 17214564

[B11] Chmielewski W. X. Beste C. (2017). Testing interactive effects of automatic and conflict control processes during response inhibition—A system neurophysiological study. NeuroImage 146, 1149–1156. 10.1016/j.neuroimage.2016.10.015 27742599

[B12] Connolly J. D. Goodale M. A. DeSouza J. F. Menon R. S. Vilis T. (2000). A comparison of frontoparietal fMRI activation during anti-saccades and anti-pointing. J. Neurophysiol. 84, 1645–1655. 10.1152/jn.2000.84.3.1645 10980034

[B13] Coxon J. P. Stinear C. M. Byblow W. D. (2006). Intracortical inhibition during volitional inhibition of prepared action. J. Neurophysiol. 95, 3371–3383. 10.1152/jn.01334.2005 16495356

[B14] Coxon J. P. Stinear C. M. Byblow W. D. (2007). Selective inhibition of movement. J. Neurophysiol. 97, 2480–2489. 10.1152/jn.01284.2006 17251361

[B15] Coxon J. P. Stinear C. M. Byblow W. D. (2009). Stop and go: the neural basis of selective movement prevention. J. Cogn. Neurosci. 21, 1193–1203. 10.1162/jocn.2009.21081 18702592

[B16] De Pretto M. Rochat L. Spierer L. (2017). Spatiotemporal brain dynamics supporting the immediate automatization of inhibitory control by implementation intentions. Sci. Rep. 7:10821. 10.1038/s41598-017-10832-x 28883497 PMC5589860

[B17] Dehaene S. Piazza M. Pinel P. Cohen L. (2003). Three parietal circuits for number processing. Cogn. Neuropsychol. 20, 487–506. 10.1080/02643290244000239 20957581

[B18] Dillon D. G. Pizzagalli D. A. (2007). Inhibition of action, thought and emotion: a selective neurobiological review. Appl. Prev. Psychol. 12, 99–114. 10.1016/j.appsy.2007.09.004 19050749 PMC2396584

[B19] Egenolf Y. Stein M. Koenig T. Grosse Holtforth M. Dierks T. Caspar F. (2013). Tracking the implicit self using event-related potentials. Cogn. Affect. Behav. Neurosci. 13, 885–899. 10.3758/s13415-013-0169-3 23636983

[B20] Enge S. Behnke A. Fleischhauer M. Küttler L. Kliegel M. Strobel A. (2014). No evidence for true training and transfer effects after inhibitory control training in young healthy adults. J. Exp. Psychol. Learn. Mem. Cogn. 40, 987–1001. 10.1037/a0036165 24707778

[B21] Enriquez-Geppert S. Konrad C. Pantev C. Huster R. J. (2010). Conflict and inhibition differentially affect the N200/P300 complex in a combined go/nogo and stop-signal task. NeuroImage 51, 877–887. 10.1016/j.neuroimage.2010.02.043 20188191

[B22] Falkenstein M. Hoormann J. Hohnsbein J. (1999). ERP components in go/nogo tasks and their relation to inhibition. Acta Psychol. 101, 267–291. 10.1016/s0001-6918(99)00008-6 10344188

[B23] Fan L.-Y. Gau S. S.-F. Chou T.-L. (2014). Neural correlates of inhibitory control and visual processing in youths with attention deficit hyperactivity disorder: a counting Stroop functional MRI study. Psycholo. Med. 44, 2661–2671. 10.1017/S0033291714000038 24451066

[B24] Fan L.-Y. Chou T.-L. Gau S. S.-F. (2017). Neural correlates of atomoxetine improving inhibitory control and visual processing in Drug-naïve adults with attention-deficit/hyperactivity disorder. Hum. Brain Mapp. 38, 4850–4864. 10.1002/hbm.23683 28657141 PMC6867141

[B25] Federico P. Perez M. A. (2017). Altered corticospinal function during movement preparation in humans with spinal cord injury. J. Physiol. 595, 233–245. 10.1113/JP272266 27485306 PMC5199749

[B26] Folstein J. R. Van Petten C. (2008). Influence of cognitive control and mismatch on the N2 component of the ERP: a review. Psychophysiology 45, 152–170. 10.1111/j.1469-8986.2007.00602.x 17850238 PMC2365910

[B27] Gajewski P. D. Falkenstein M. (2013). Effects of task complexity on ERP components in go/nogo tasks. Int. J. Psychophysiol. 87, 273–278. 10.1016/j.ijpsycho.2012.08.007 22906814

[B28] Ganos C. Rothwell J. Haggard P. (2018). Voluntary inhibitory motor control over involuntary tic movements. Mov. Disord. 33, 937–946. 10.1002/mds.27346 29508917

[B29] Habermann M. Weusmann D. Stein M. Koenig T. (2018). A student’s guide to randomization statistics for multichannel event-related potentials using ragu. Front. Neurosci. 12:355. 10.3389/fnins.2018.00355 29973861 PMC6020783

[B30] Hartmann L. Sallard E. Spierer L. (2016). Enhancing frontal top-down inhibitory control with go/nogo training. Brain Struct. Funct. 221, 3835–3842. 10.1007/s00429-015-1131-7 26459141

[B31] Hartmann L. Wachtl L. de Lucia M. Spierer L. (2019). Practice-induced functional plasticity in inhibitory control interacts with aging. Brain Cogn. 132, 22–32. 10.1016/j.bandc.2019.02.004 30802731

[B32] Heinen S. J. Rowland J. Lee B.-T. Wade A. R. (2006). An oculomotor decision process revealed by functional magnetic resonance imaging. J. Neurosci. 26, 13515–13522. 10.1523/JNEUROSCI.4243-06.2006 17192434 PMC6674715

[B33] Herweg N. A. Weber B. Kasparbauer A. Meyhöfer I. Steffens M. Smyrnis N.. (2014). Functional magnetic resonance imaging of sensorimotor transformations in saccades and antisaccades. NeuroImage 102, 848–860. 10.1016/j.neuroimage.2014.08.033 25173413

[B34] Huestegge L. Koch I. (2014). When two actions are easier than one: How inhibitory control demands affect response processing. Acta Psychol. 151, 230–236. 10.1016/j.actpsy.2014.07.001 25086224

[B35] Jamadar S. Fielding J. Egan G. (2013). Quantitative meta-analysis of fMRI and PET studies reveals consistent activation in fronto-striatal-parietal regions and cerebellum during antisaccades and prosaccades. Front. Psychol. 4:749. 10.3389/fpsyg.2013.00749 24137150 PMC3797465

[B36] Koenig T. Melie-García L. (2010). A method to determine the presence of averaged event-related fields using randomization tests. Brain Topogr. 23, 233–242. 10.1007/s10548-010-0142-1 20376546

[B37] Koenig T. Kottlow M. Stein M. Melie-García L. (2011). Ragu: A free tool for the analysis of EEG and MEG event-related scalp field data using global randomization statistics. Comput. Intell. Neurosci. 2011:938925. 10.1155/2011/938925 21403863 PMC3049349

[B38] Kok A. Ramautar J. R. De Ruiter M. B. Band G. P. H. Ridderinkhof K. R. (2004). ERP components associated with successful and unsuccessful stopping in a stop-signal task. Psychophysiology 41, 9–20. 10.1046/j.1469-8986.2003.00127.x 14692996

[B39] Lévesque J. Beauregard M. Mensour B. (2006). Effect of neurofeedback training on the neural substrates of selective attention in children with attention-deficit/hyperactivity disorder: a functional magnetic resonance imaging study. Neurosci. Lett. 394, 216–221. 10.1016/j.neulet.2005.10.100 16343769

[B40] Leung H.-C. Cai W. (2007). Common and differential ventrolateral prefrontal activity during inhibition of hand eye movements. J. Neurosci. 27, 9893–9900. 10.1523/JNEUROSCI.2837-07.2007 17855604 PMC6672638

[B41] Liu H. Liang L. Dunlap S. Fan N. Chen B. (2016). The effect of domain-general inhibition-related training on language switching: an ERP study. Cognition 146, 264–276. 10.1016/j.cognition.2015.10.004 26491833

[B42] Lucci G. Pisotta I. Berchicci M. Di Russo F. Bonavita J. Scivoletto G.. (2019). Proactive cortical control in spinal cord injury subjects with paraplegia. J. Neurotrauma 36, 3347–3355. 10.1089/neu.2018.6307 31017041

[B43] Lundstrom B. N. Ingvar M. Petersson K. M. (2005). The role of precuneus and left inferior frontal cortex during source memory episodic retrieval. NeuroImage 27, 824–834. 10.1016/j.neuroimage.2005.05.008 15982902

[B44] Manuel A. L. Bernasconi F. Spierer L. (2013). Plastic modifications within inhibitory control networks induced by practicing a stop-signal task: an electrical neuroimaging study. Cortex 49, 1141–1147. 10.1016/j.cortex.2012.12.009 23313010

[B45] Maraver M. J. Bajo M. T. Gomez-Ariza C. J. (2016). Training on working memory and inhibitory control in young adults. Front. Hum. Neurosci. 10:588. 10.3389/fnhum.2016.00588 27917117 PMC5114277

[B46] McDowell J. E. Dyckman K. A. Austin B. P. Clementz B. A. (2008). Neurophysiology and neuroanatomy of reflexive and volitional saccades: evidence from studies of humans. Brain Cogn. 68, 255–270. 10.1016/j.bandc.2008.08.016 18835656 PMC2614688

[B47] Michel C. Koenig T. Brandeis D. (2009). “Electrical neuroimaging in the time domain,” in Electrical Neuroimaging, eds Michel C. Koenig T. Brandeis D. Gianotti L. Wackermann J. (Cambridge: Cambridge University Press), 111–144.

[B48] Millner A. J. Jaroszewski A. C. Chamarthi H. Pizzagalli D. A. (2012). Behavioral and electrophysiological correlates of training-induced cognitive control improvements. NeuroImage 63, 742–753. 10.1016/j.neuroimage.2012.07.032 22836178 PMC3601637

[B49] Miyake A. Friedman N. P. Emerson M. J. Witzki A. H. Howerter A. Wager T. D. (2000). The unity and diversity of executive functions and their contributions to complex “frontal lobe” tasks: a latent variable analysis. Cogn. Psychol. 41, 49–100. 10.1006/cogp.1999.0734 10945922

[B50] Molenberghs P. Mesulam M. M. Peeters R. Vandenberghe R. R. C. (2007). Remapping attentional priorities: differential contribution of superior parietal lobule and intraparietal sulcus. Cereb. Cortex 17, 2703–2712. 10.1093/cercor/bhl179 17264251

[B51] Murray M. M. Brunet D. Michel C. M. (2008). Topographic ERP analyses: a step-by-step tutorial review. Brain Topogr. 20, 249–264. 10.1007/s10548-008-0054-5 18347966

[B52] Neggers S. F. W. van Diepen R. M. Zandbelt B. B. Vink M. Mandl R. C. W. Gutteling T. P. (2012). A functional and structural investigation of the human fronto-basal volitional saccade network. PLoS One 7:e29517. 10.1371/journal.pone.0029517 22235303 PMC3250458

[B53] Olman C. A. Pickett K. A. Schallmo M.-P. Kimberley T. J. (2012). Selective BOLD responses to individual finger movement measured with fMRI at 3T. Hum. Brain Mapp. 33, 1594–1606. 10.1002/hbm.21310 21674691 PMC3713710

[B54] Pascual-Marqui R. D. (2002). Standardized low-resolution brain electromagnetic tomography (sLORETA): technical details. Methods Find. Exp. Clin. Pharmacol. 24, 5–12. 12575463

[B55] Pascual-Marqui R. D. (1999). Review of methods for solving the EEG inverse problem. Int. J. Bioelectromagn. 1, 75–86.

[B56] Perrin F. Pernier J. Bertrand O. Giard M. H. Echallier J. F. (1987). Mapping of scalp potentials by surface spline interpolation. Electroencephalogr. Clin. Neurophysiol. 66, 75–81. 10.1016/0013-4694(87)90141-6 2431869

[B57] Popov T. Westner B. U. Silton R. L. Sass S. M. Spielberg J. M. Rockstroh B.. (2018). Time course of brain network reconfiguration supporting inhibitory control. J. Neurosci. 38, 4348–4356. 10.1523/JNEUROSCI.2639-17.2018 29636394 PMC5932643

[B58] Rey-Mermet A. Gade M. Oberauer K. (2018). Should we stop thinking about inhibition? Searching for individual and age differences in inhibition ability. J. Exp. Psychol. Learn. Mem. Cogn. 44, 501–526. 10.1037/xlm0000450 28956944

[B59] Rubia K. Russell T. Overmeyer S. Brammer M. J. Bullmore E. T. Sharma T.. (2001). Mapping motor inhibition: conjunctive brain activations across different versions of go/no-go and stop tasks. NeuroImage 13, 250–261. 10.1006/nimg.2000.0685 11162266

[B60] Ruggeri P. Meziane H. B. Koenig T. Brandner C. (2019). A fine-grained time course investigation of brain dynamics during conflict monitoring. Sci. Rep. 9:3667. 10.1038/s41598-019-40277-3 30842528 PMC6403345

[B61] Sallard E. Barral J. Chavan C. F. Spierer L. (2014). Early attentional processes distinguish selective from global motor inhibitory control: An electrical neuroimaging study. NeuroImage 87, 183–189. 10.1016/j.neuroimage.2013.11.002 24220039

[B62] Scharinger C. Soutschek A. Schubert T. Gerjets P. (2015). When flanker meets the n-back: what EEG and pupil dilation data reveal about the interplay between the two central-executive working memory functions inhibition and updating. Psychophysiology 52, 1293–1304. 10.1111/psyp.12500 26238380

[B63] Schiller B. Gianotti L. R. R. Baumgartner T. Nash K. Koenig T. Knoch D. (2016). Clocking the social mind by identifying mental processes in the IAT with electrical neuroimaging. Proc. Natl. Acad. Sci. U S A 113, 2786–2791. 10.1073/pnas.1515828113 26903643 PMC4791005

[B64] Senkowski D. Herrmann C. S. (2002). Effects of task difficulty on evoked gamma activity and ERPs in a visual discrimination task. Clin. Neurophysiol. 113, 1742–1753. 10.1016/s1388-2457(02)00266-3 12417227

[B65] Simonet M. von Roten F. C. Spierer L. Barral J. (2019). Executive control training does not generalize, even when associated with plastic changes in domain-general prefrontal areas. NeuroImage 197, 457–469. 10.1016/j.neuroimage.2019.04.010 30974240

[B66] Smith J. L. Johnstone S. J. Barry R. J. (2006). Effects of pre-stimulus processing on subsequent events in a warned Go/NoGo paradigm: response preparation, execution and inhibition. Int. J. Psychophysiol. 61, 121–133. 10.1016/j.ijpsycho.2005.07.013 16214250

[B67] Spielberg J. M. Miller G. A. Heller W. Banich M. T. (2015). Flexible brain network reconfiguration supporting inhibitory control. Proc. Natl. Acad. Sci. U S A 112, 10020–10025. 10.1073/pnas.1500048112 26216985 PMC4538617

[B68] Spierer L. Chavan C. F. Manuel A. L. (2013). Training-induced behavioral and brain plasticity in inhibitory control. Front. Hum. Neurosci. 7:427. 10.3389/fnhum.2013.00427 23914169 PMC3729983

[B69] Strik W. K. Fallgatter A. J. Brandeis D. Pascual-Marqui R. D. (1998). Three-dimensional tomography of event-related potentials during response inhibition: evidence for phasic frontal lobe activation. Electroencephalogr. Clin. Neurophysiol. 108, 406–413. 10.1016/s0168-5597(98)00021-5 9714383

[B70] Swick D. Ashley V. Turken A. U. (2008). Left inferior frontal gyrus is critical for response inhibition. BMC Neurosci. 9:102. 10.1186/1471-2202-9-102 18939997 PMC2588614

[B71] Talanow T. Kasparbauer A.-M. Lippold J. V. Weber B. Ettinger U. (2018). Neural correlates of proactive and reactive inhibition of saccadic eye movements. Brain Imaging Behav. 14, 72–88. 10.1007/s11682-018-9972-3 30298238

[B72] Verbruggen F. Adams R. Chambers C. D. (2012). Proactive motor control reduces monetary risk taking in gambling. Psychol. Sci. 23, 805–815. 10.1177/0956797611434538 22692336 PMC3724270

[B73] Vernet M. Quentin R. Chanes L. Mitsumasu A. Valero-Cabré A. (2014). Frontal eye field, where art thou? Anatomy, function and non-invasive manipulation of frontal regions involved in eye movements and associated cognitive operations. Front. Int. Neurosci. 8:66. 10.3389/fnint.2014.00066 25202241 PMC4141567

[B74] Vuillier L. Bryce D. Szücs D. Whitebread D. (2016). The maturation of interference suppression and response inhibition: ERP analysis of a cued go/nogo task. PLoS One 11:e0165697. 10.1371/journal.pone.0165697 27814356 PMC5096696

[B75] Wenderoth N. Debaere F. Sunaert S. Swinnen S. P. (2005). The role of anterior cingulate cortex and precuneus in the coordination of motor behaviour. Eur. J. Neurosci. 22, 235–246. 10.1111/j.1460-9568.2005.04176.x 16029213

[B76] Wessel J. R. Aron A. R. (2017). On the globality of motor suppression: unexpected events and their influence on behavior and cognition. Neuron 93, 259–280. 10.1016/j.neuron.2016.12.013 28103476 PMC5260803

[B77] Witt S. T. Meyerand M. E. Laird A. R. (2008). Functional neuroimaging correlates of finger tapping task variations: an ALE meta-analysis. NeuroImage 42, 343–356. 10.1016/j.neuroimage.2008.04.025 18511305 PMC2592684

